# Atomic-Scale Insights into Surface Reconstruction and Dissolution of Hematite: The Formation of Water Cages and Protonation Effects

**DOI:** 10.3390/molecules31040748

**Published:** 2026-02-22

**Authors:** Wenjie Zhou, Chaofang Dong

**Affiliations:** Key Laboratory of Corrosion and Protection (Ministry of Education), Institute for Advanced Materials and Technology, University of Science and Technology Beijing, Beijing 100083, China

**Keywords:** dissolution mechanism, ab initio molecular dynamics, metadynamics simulations, surface reconstruction, hydrogen-bonding net

## Abstract

Dissolution of iron oxides in water plays a critical role in corrosion, mineral cycling, and surface reactivity; yet, the atomic-scale mechanisms governing Fe release remain poorly understood. Here, we employ ab initio molecular dynamics and well-tempered metadynamics simulations to investigate the stepwise dissolution of surface Fe atoms from the -Fe_2_O_3_(0001) surface in aqueous solution. The dissolution process initiates from a stable surface configuration in which Fe is coordinated to three lattice oxygen atoms and one water molecule. It proceeds through a series of metastable states involving additional water coordination, proton-assisted Fe-O bond weakening, and eventual detachment from the substrate. The first major transition, requiring 46.5 kJ/mol, involves breaking the hydrogen-bonding net and overcoming steric hindrance to allow adsorption of a second water molecule. Intermediate barriers (10.9–30.3 kJ/mol) are associated with further coordination and bond cleavage steps. In contrast, the final release of Fe into the solution, corresponding to a state coordinated with four water molecules and no lattice oxygen, exhibits a much higher free-energy barrier of ~93.0 kJ/mol. This barrier arises from the formation of a rigid hydrogen-bonded water cage and the loss of proton access to the remaining surface oxygen site, as confirmed by radial distribution function analysis. Our findings reveal why -Fe_2_O_3_(0001) is highly resistant to complete dissolution yet prone to surface roughening, defect formation, and adatom structures under aqueous conditions.

## 1. Introduction

Material dissolution is a ubiquitous process that permeates numerous scientific fields, exerting both advantageous and deleterious effects on a wide array of phenomena. Probing the atomic-level microscopic mechanisms underlying this process remains a core focus in many research fields, such as geochemistry [1,2], corrosion of metals and alloys [3], degradation of catalysts [4], and drug release [5]. Hematite, which is also known as α-Fe_2_O_3_, is one of the most common materials and is abundantly found in soils, sediments, and natural water [6,7]. Owing to its low cost, high stability, and non-toxicity, hematite has been widely used as a primary component of coating sands in wastewater treatment [8]. Its narrow bandgap characteristics have also garnered attention in the field of photocatalytic water splitting [9,10]. In addition, as a prominent component of the outermost layer of passivation films on iron-based materials, an extensive array of DFT studies has delved into the properties of these films by modeling the interaction of hematite with various corrosive ions and defects [11,12].

Even though hematite inherently possesses high stability, it still faces potential dissolution risks in various application scenarios. For instance, defects are designed on the hematite surface to accelerate photo-induced water splitting [13,14,15]. The size of hematite in most catalyst application scenarios is designed into the nanoscale [16,17,18]. Furthermore, in certain service environments of stainless steel, such as Proton Exchange Membrane Fuel Cells (PEMFC) [19,20,21] and flue gas desulfurization [19], hematite, being the primary constituent of the passive film on the surface, is directly exposed to high temperatures and strong acidic environments. Thus, understanding the mechanism of the dissolution process is also essential for hematite.

In general, the dissolution rate is determined by the temperature, the strength of illumination, the composition of the solution phase, the specific surface area, the particle size, and the presence of defects or the steps on the surface [22,23]. Theoretically, one of the significant driving forces of the dissolution reaction is undersaturation [24]. Numerous studies have reported a phenomenon where the dissolution rate of hematite is initially very rapid and subsequently decreases sharply [25,26].

Proton-promoted, ligand-promoted, and (photo)reductive dissolution are widely recognized as the main pathways of dissolution [27,28]. However, these theories primarily stem from conjectures based on macroscopic experimental results, thereby lacking the capacity to explain more microscopic details, such as “more dimples and bumps” on the surface away from the steps [29]. With the continual advancement of technology, an increasing number of techniques, such as Crystal Truncation Rod (CTR) and Liquid Cell Transmission Electron Microscopy (LCTEM) [30,31], have been developed to observe the hematite–water interface at a microscopic scale. However, due to the difficulty of extending the time scale, these techniques only provided limited insights into the interface kinetic process [32,33,34].

Atomic simulation serves as an invaluable tool in offering an atomistic perspective for understanding complex scientific phenomena. Until now, numerous studies [35,36,37,38] have reported the micro details of hematite–water interfaces via ab initio molecular dynamics (AIMD). However, given that bond formation and cleavage during dissolution are rare events on the timescale accessible to AIMD, these studies have not exhibited a tendency towards dissolution. Until now, the detailed processes at the atomic level involved in hematite dissolution remain a void in our current studies. Metadynamics [39,40], one of the enhancing sampling methods, can apply a bias potential on the selected collective variables (CVs) to explore a broader phase space of the system. It has thus become a powerful method for exploring the detailed process of dissolution. Since the bond formation and scissions are inevitable in the dissolution process, the classical force field is not capable of explaining this phenomenon. F Uddin et al. [41] employed a combination of reactive force field (ReaxFF) and metadynamics to compute the free-energy changes associated with the dissolution of Ca ions from various Ca_3_SiO_5_ surfaces along the CV of distance. Felix et al. [42] also used ReaxFF and metadynamics to investigate the dissolution mechanisms, as well as the further effects caused by crack stress. However, even though ReaxFF adds distance-dependent bond order to simulate the covalent interactions, the chosen CVs cannot efficiently describe the phase space for the dissolution process. Li et al. [43] employed CVs of coordinate number (CN) for the dissolution of Ca_3_SiO_5_ to describe the process at the ab initio level. Réocreux et al. [44] also calculate the free-energy landscape in CVs of CN for the dissolution process of Al_2_O_3_ by ab initio metadynamics. Klyukun et al. [45] also studied the dissolution kinetics of goethite from different facets. However, these results focused primarily on the free-energy landscape brought by metadynamics, overlooking the fact that ab initio level calculations can also give detailed electronic structures for these processes, which can enhance our understanding of the process.

In this study, the dissolution mechanism of hematite in the early stage has been uncovered. We employed AIMD combined with metadynamics to compute the free-energy landscape and thereby the reaction pathway for a ferric ion detachment and subsequent decomposition from the Fe-terminated hydroxylation α-Fe_2_O_3_(0001). The results are in proper agreement with the ligand-promoted and proton-promoted mechanism. It shows that interfacial water molecules act as ligands to facilitate the detachment of ferric ions from the surface, while hydrogen adsorption weakens the Fe-O bond. Moreover, the hydrogen atom adsorbed on the oxygen near the detached iron will enter the original iron site. The steric repulsion between them and the iron atom will further reduce the free-energy barrier of dissolution. IRI (IGMH) analysis indicates that the hydrogen entering this site will form an intramolecular hydrogen bond with nearby surface oxygen, thereby stabilizing the hydrogen at this site. In the further deposition process, due to the repulsion of the hydrogen, the defect site of the leaching point, which should have a higher deposition priority, is not considered first. Our findings elucidate scenarios where the surface exhibits numerous protrusions and recesses, which traditional theories fail to account for. We have also discovered another crucial role of hydrogen atoms in the dissolution process. These insights offer further atomistic understanding of the dissolution and reconstruction of the hematite surface.

## 2. Results and Discussion

### 2.1. AIMD for Pre-Equilibrium

**Figure 1:**
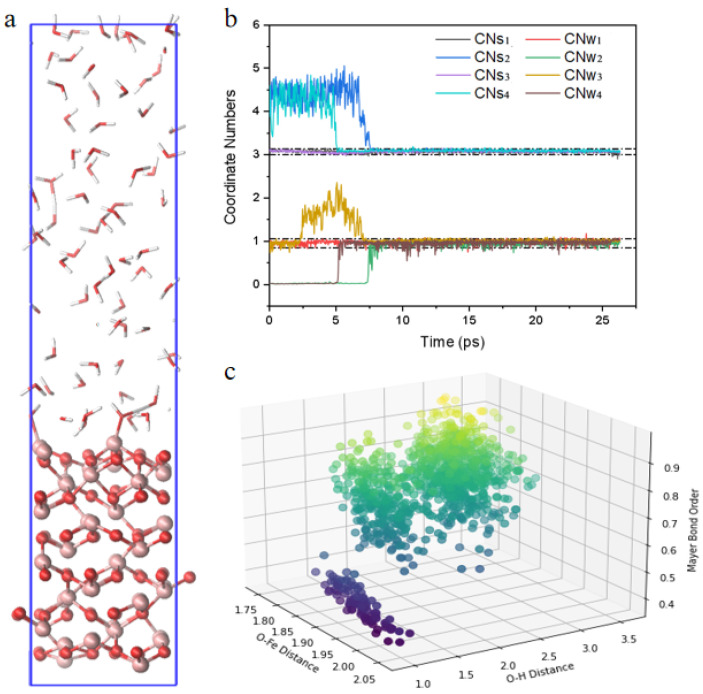
(**a**) The initial simulation model used in AIMD calculations consists of an α-Fe_2_O_3_(0001) slab (bottom) in contact with an explicit water layer (top). The blue box indicates the periodic simulation cell; (**b**) Time evolution of coordination numbers for four representative surface Fe atoms; (**c**) The relationship between Mayer bond order of the Fe-O bond and the corresponding Fe-O and O-H distances. The color gradient represents the magnitude of the Mayer bond order, ranging from dark blue (weaker Fe-O bonds) to yellow (stronger Fe-O bonds).

To establish reasonable system parameters and gather relevant data, we conducted a 25-ps AIMD simulation on the system depicted in Figure 1. Additionally, we conducted a comprehensive analysis of the overall atomic density (Supplementary Materials Figure S1), which closely matched a water solution concentration of approximately 55.5 mol/L, as indicated by the dashed line. This confirms the validity of our system setup.

Furthermore, single-point energy calculations were performed every 0.25 ps to further investigate the relationships between Fe-O bond Mayer bond orders and O-Fe and O-H bonds (Figure 1c). We observed a gradual reduction in the Mayer bond order of the O-Fe bond as the distance increased. Notably, a significant decrease in the Mayer bond order of the O-Fe bond occurred when the distance to neighboring O-H bonds decreased to approximately 1.2 Å. This sharp decrease underscores the substantial weakening effect of hydrogen atoms on the O-Fe bond energy, aligning with theories of proton-induced dissolution.

While hydrogen atoms effectively reduce the strength of O-Fe bonds, the amplitude of CNs is not large enough. Observing dissolution phenomena within the simulated AIMD timeframe remains challenging. Therefore, we proceeded with metadynamics simulations to explore this further.

### 2.2. Dissolution Pathway for Fe from (3,1) to (1,4)

**Figure 2:**
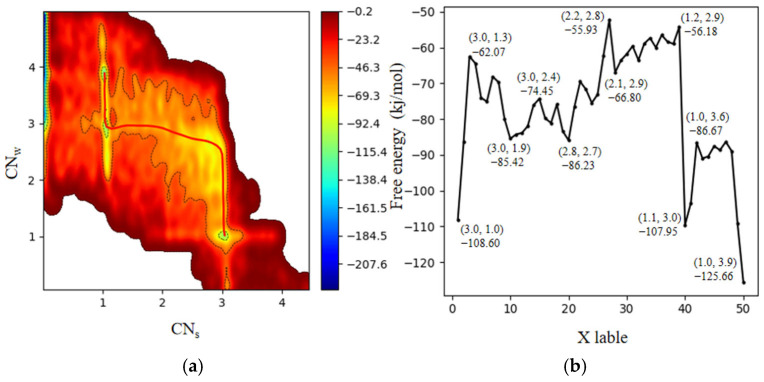
(**a**) Two-dimensional free-energy surface (FES) of Fe dissolution from the α-Fe_2_O_3_(0001) surface with variables of CNs and CNw; (**b**) One-dimensional free-energy profile extracted along the minimum energy path is shown in the red line in panel (**a**).

To elucidate the dissolution mechanism of surface Fe atoms, we performed an extended 70-ps well-tempered metadynamics simulation. As illustrated in Figure 2a, CNs and CNw are used as the x-axis and y-axis, respectively, to describe the state of Fe atoms from the matrix to the dissolution process. In the CVs-times plot provided in the Supplementary Information, the CVs oscillated within the same basin, indicating that the bias potential has effectively filled the basin and that the simulation has reached a well-converged state. As a result, the added bias allows for accurate reconstruction of the underlying free-energy surface. The obtained potential energy surface effectively captures the dynamic processes at the surface.

After obtaining the FES, the minimum free-energy pathway connecting the initial and final states—corresponding to the transition from a (3,1) to a (1,4) coordination environment—was identified using a pathway search algorithm, MEPplot. The resulting pathway is highlighted as a red line in Figure 2a. Furthermore, the free-energy profile along the minimum energy path was extracted and is presented in Figure 2b. This profile clearly reveals the presence of intermediate metastable states and the corresponding energy barriers between different configurations along the dissolution pathway. These results offer mechanistic insight into the stepwise kinetics of surface Fe atom dissolution.

**Figure 3:**
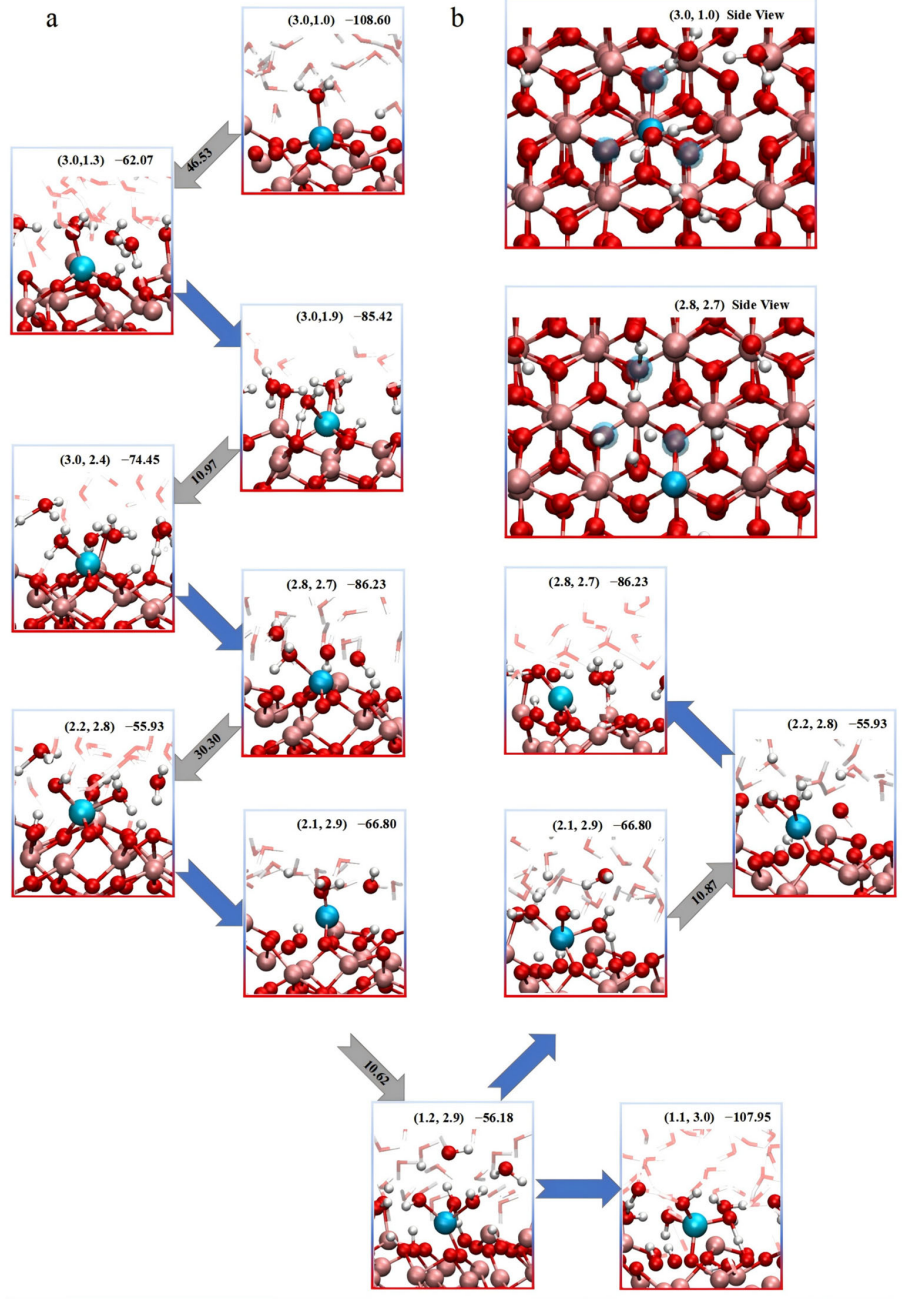
(**a**) Key intermediate configurations along the dissolution pathway from the α-Fe_2_O_3_(0001). Each structure is labeled with its corresponding coordination numbers (CN, CNw) and relative free-energy (kJ/mol); the values in the arrows in gray are the free-energy barriers (kJ/mol) from different configurations. In all structure snapshots, the cyan spheres represent the dissolving Fe atom, while red and white spheres correspond to oxygen and hydrogen atoms, respectively. The bulk Fe atoms within the hematite lattice are shown in pink, indicating their immobile, lattice-coordinated nature. For clarity, water molecules are rendered in stick representation in some panels to emphasize interaction networks, such as hydrogen bonding. (**b**) The side view of the configuration in which state at (3.0,1.0) and (2.8,2.7), The oxygen atoms originally coordinated with the dissolving Fe atom are highlighted with semi-transparent blue spheres. The dissoluted Fe have already migration into an add-atom state.

We further extracted the free-energy values and corresponding configurations at each critical point, which allowed us to construct a clearer depiction of the path transitions, as shown in Figure 3a. During the early stages of dissolution, the transition from the (3,1) to the (3,1.3) configuration corresponds to the first significant energy barrier. This state involves the adsorption of a second water molecule in close proximity to the Fe atom. The IRI analysis, provided in the (Supplementary Materials), reveals that the approach of the additional water molecule must overcome dual steric hindrance from both the surface oxygen of the matrix and the adsorbed oxygen. Moreover, in this configuration, two oxygen atoms move closer together, thereby disrupting the hydrogen-bond network at the surface. This indicates that, at this point, not only must the system overcome steric hindrance from the surface, but it must also break the pre-formed hydrogen-bonding network established during surface adsorption.

It is worth noting that this transition represents the rate-determining step in the entire dissolution pathway from the initial (3,1) to the final (1,4) coordination state. This step exhibits the highest free-energy barrier of 46.53 kJ/mol. Once this state is reached, subsequent approach and adsorption of the oxygen atom toward the Fe center, leading to the (3,1.9) configuration, proceed spontaneously without additional energy input. The next energy barrier occurs during the transition from the (3,1.9) adsorbed state to the (3,2.4) state, where another oxygen atom approaches the Fe atom. However, this configuration is relatively unstable due to the difficulty in forming a compact hydrogen-bonding network at the surface. As such, the newly approaching oxygen atom needs only to overcome local steric hindrance to coordinate with Fe. The associated energy barrier for this step is relatively low, at 10.97 kJ/mol. Following the successful adsorption of three water molecules, the system stabilizes at the (2.8,2.7) state. This indicates that further coordination by aqueous-phase oxygen atoms weakens the remaining Fe-O bonds within the substrate. At this stage, the Fe-O bond lengths in the lattice are noticeably elongated, confirming progressive weakening of the Fe anchoring within the hematite framework.

The subsequent step involves a bond-breaking event in which the Fe atom detaches from one of its coordinating lattice oxygen atoms, transitioning from a six-fold coordination environment into the (2.2,2.8) state and initiating its progressive release into the solution. This step is associated with a moderate energy barrier of 30.30 kJ/mol. Analysis of several structural snapshots corresponding to similar configurations consistently revealed that the dissociating oxygen atoms are protonated. This observation provides further evidence supporting the proton-assisted dissolution mechanism, wherein protonation of surface oxygen weakens the Fe-O bonds and facilitates their cleavage. Following this bond-breaking step, the dissolution proceeds through additional weakening of Fe-O bonds and further coordination with water molecules, driving the Fe atom closer to a fully solvated state.

Interestingly, during the final stages of the dissolution process, although no significant energy barrier is observed along the pathway, structural analysis of the associated configurations reveals that the Fe atom does not proceed directly into the bulk solution. Instead, it undergoes a fluctuating motion and reattaches to the surface, forming a new add-atom configuration. As shown in the lower branch of Figure 3a, the Fe atom transitions from a (1,3) state back to a (2.2,2.8) configuration by re-adsorbing onto the surface. Further insights were obtained from the top-down view in Figure 3b, which shows that the new add-atom site adopted by the Fe atom coincides with the lattice site of a second-layer Fe atom in the underlying hematite structure. This preferred reattachment site suggests a structural registry with the bulk lattice. The tendency for the Fe atom to form an add-atom configuration rather than returning to its original lattice site appears to be driven by the presence of a surface Fe vacancy. Once the Fe atom detaches from the surface, the resulting negatively charged defect tends to attract and bind protons from the surrounding aqueous environment. As shown in Figure 3b, the Fe vacancy corresponding to the (2.2,2.8) configuration is already occupied by two protons. The proton occupation of the vacancy site effectively blocks the Fe atom from reoccupying its original position, thus forcing it to migrate to an adjacent coordination site where it stabilizes as an add-atom. This mechanism provides a plausible explanation for the experimentally observed formation of uneven, defect-rich surfaces during iron oxide dissolution.

To further evaluate the stability of protonated configurations at Fe vacancy sites, we randomly selected two representative structures featuring dual hydrogen adsorption at the vacancy for subsequent AIMD simulations and IRI analyses. As shown in the Supplementary Information, both configurations exhibit excellent structural stability, with angular deviations of the adsorbed hydrogen atoms remaining within 1.0° and bond length fluctuations limited to less than 0.1 Å throughout the trajectory. IRI analysis reveals that each proton forms a well-defined intramolecular hydrogen bond with a neighboring lattice oxygen atom directly opposite the vacancy. This interaction significantly enhances the overall stability of the system and effectively anchors the protons within the Fe vacancy.

### 2.3. Complete Dissolution and Water Cages

**Figure 4:**
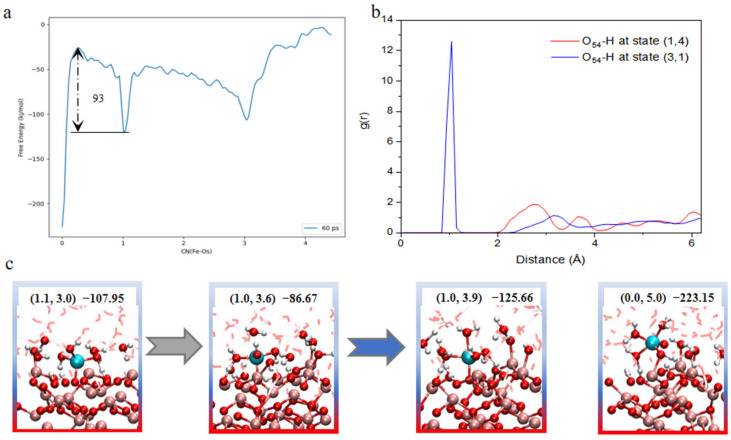
(**a**) One-dimensional projection of the free-energy surface (FES) along with the variables CNs. (**b**) Radial distribution function (RDF) analysis of the selective terminal lattice oxygen (O54) with respect to hydrogen atoms (H) at two representative states: (3,1) and (1,4). (**c**) Representative configurations along the final stage of Fe dissolution, progressing from state (1.1,3.0) to the fully solvated state (0.0,5.0).

Although previous simulations successfully captured a series of intermediate configurations during the dissolution process of surface Fe atoms, the system still remains far from achieving complete detachment. Furthermore, direct pathway searches failed to identify a well-defined minimum energy path leading to full dissolution. To estimate the energy requirement for this process, we projected the two-dimensional free-energy surface onto a one-dimensional coordinate, as shown in Figure 4a. From this projection, the estimated free-energy barrier for complete dissolution is approximately 93 kJ/mol.

Representative configurations corresponding to near-complete and fully dissociated states are shown in Figure 4c. These structures reveal the key reason why breaking the final Fe-O bond is significantly more difficult than earlier stages. Once the system reaches the (1,3) coordination state, the water molecules coordinated to Fe tend to form strong hydrogen bonds between their hydrogen atoms and the lattice oxygen atoms. In parallel, any water molecules potentially adsorbed on the top surface become integrated into the extended hydrogen-bonding network of the bulk water, leading to the formation of a rigid water cage surrounding the Fe center. Within this cage, most of the surrounding water molecules participate in stable hydrogen bonds, meaning that the final detachment of Fe requires the simultaneous disruption of multiple hydrogen bonds. This energy demand is substantially higher than earlier steps, such as the (3,1) to (3,1.3) transition, which involves breaking only a single adsorbed water molecule from the hydrogen-bonding net. Additionally, the formation of the water cage prevents further protonation of the lattice oxygen to which the Fe remains bonded. Radial distribution function analysis of the specific O-H distances for configurations near (1,4) compared to those near (3,1) reveals that the characteristic peak corresponding to proton adsorption vanishes entirely in the former. This indicates that the terminal oxygen atoms become inaccessible to protons in the late stages of dissolution. As a result, the final bond cleavage must occur without the benefit of proton-induced bond weakening. These two effects induced by the formation of the water cage account for the remarkably high free-energy requirement observed in this stage.

## 3. Materials and Methods

### 3.1. Atomistic Model

Given the high likelihood of formation and exceptional catalytic characteristics of the Fe_2_O_3_(0001) surface, it was selected as the focus of our investigation. A pure surface model was procured from our existing studies. To facilitate upcoming dynamic simulations, this was subsequently transformed into an orthogonal lattice expanded by a factor of $\sqrt{3}\;*\;2$. Considering the high turnover frequency of adsorbed water molecules at the Fe_2_O_3_(0001)/water interface [34], it is unnecessary to pre-set these molecules, as they are expected to spontaneously adsorb during dynamic equilibration. Subsequently, a water box was constructed, encapsulating 69 water molecules at a density of 1 mg/L, and featuring lattice dimensions that are commensurate with the solid component. Both surfaces were kept in direct contact with the water molecules. The resultant model (totally 327 atoms) had lattice parameters of 10.21 Å × 8.84 Å × 40.27 Å. After conducting AIMD simulations for a period of 5 ps, substantial equilibrium within the model was achieved, thereby facilitating a more thorough exploration of its equilibrium properties and dissolution characteristics. Regarding the choice of the specific site in this work, our motivation was partly inspired by the experimental observations of Eggleston et al. [23,29], who noted that even atomically flat hematite surfaces undergo significant morphological changes—developing “dimples and bumps” (surface reconstruction)—after exposure to high-temperature acidic environments. To elucidate the underlying mechanism of this phenomenon, we specifically chose to model the dissolution/reconstruction of an atom on a flat terrace site. By focusing on this site, we were able to observe how individual atoms initiate movement from the matrix and why they tend to reconstruct rather than fully dissolve under certain conditions.

### 3.2. AIMD Simulation

All the AIMD simulations reported in this work were performed within the framework of DFT with the generalized gradient approximation (GGA) using the Perdew–Burke–Ernzerhof (PBE) [46] functional and Grimme D3 correction [47], which was implemented in the CP2K/Quickstep code (Version 8.1) [48]. The core electrons were described by Goedecker–Teter–Hutter (GTH) pseudopotentials [49,50] and the valence electrons were described by a mixed Gaussian and plane wave basis (GPW) [51]. The wave functions were expanded on a double-ζ valence polarized (DZVP) basis set, along with an auxiliary plane wave basis set at a cutoff energy of 500 Ry. The orbital transformation (OT) method [52] was employed as the self-consistent field method. The Brillouin zone was sampled by the gamma approximation. During AIMD, the nuclei were treated within the Born–Oppenheimer approximation with a timestep of 0.5 fs for equilibrium simulation. The temperature of the simulations was maintained at 298 K using the Canonical Sampling through Velocity Rescaling (CSVR) thermostat [53] coupled to the system with a time constant of 200 fs in the Canonical ensemble (NVT). The convergence criterion for energy was set to 10^−11^ Hartree, and for the self-consistent field, it was set to 10^−5^ Hartree.

### 3.3. Metadynamics Simulation and MEP Search

For the well-tempered metadynamics simulation [54], we utilized two-dimensional collective variables (CV_s_) characterized by the coordination number (CN) to monitor the dissolution process. The first one (CN_s_) is the coordination number of the Fe ion with all the oxygen atoms in the hematite phase, while the second one (CN_w_) is the coordination number of the Fe ion with other oxygen atoms from the water molecules. The definition of coordination numbers is expressed by default PLUMED code [55] as follows:

(1) $$CN\left(Fe,O_{W/S}\right)=\sum_{j\in O_{W/S}}s_{ij}\left(r_{ij}\right)=\sum_{j\in O_{W/S}}\frac{1-{\left(\frac{r_{ij}-d_{0}}{r_{0}}\right)}^{n}}{1-{\left(\frac{r_{ij}-d_{0}}{r_{0}}\right)}^{m}}$$

where $r_{ij}$ is the distance between atom i and atom j, $s_{ij}\left(r_{ij}\right)$ is the switching function describing the coordination between atom i and j. $d_{0}$ is the center of the switching function ($s_{ij}\left(d_{0}\right)=1$). $r_{0}$ is the acceptance distance of the switching function, coupled with the n/m ratio, which controls at which distance the O atoms are no longer considered as bonded to atom i($s_{ij}\left(d_{0}+r_{0}\right)=\frac{n}{m}$). Here, $d_{0}$ is chosen to match the Fe-O equilibrium distance (1.6 Å). A ratio of 2/5 (n = 4, m = 10) and a $r_{0}$ of 0.9 Å has been chosen. The definition of coordination number has been further applied to the AIMD simulations to validate the coordination number in the equilibrium state.

The Gaussian hills were deposited every 20 timesteps with the initial height of 2.4 kJ/mol. Before reaching the first free-energy basins, the width of the hills was set to 0.05 for CN_s_ and 0.1 for CN_w_ from the vibration of CN in AIMD. After that, the width of CN_s_ was set to 0.1 to accelerate the filling speed.

To determine the MEP, the HILLS file generated from metadynamics was post-processed using the metadyn suite to reconstruct the multi-dimensional free-energy surface (FES) and identify local minima (basins). The MEP was then optimized using the MEPplot tool based on a string-like method. Specifically, the initial path was defined by selecting nine representative extrema points as ‘beads’ to guide the initial guess. A total of 50 images (beads) were employed to discretize the transition path. The optimization was performed with a step size of 0.003 and a maximum of 128 iterations to ensure the path converged to the true minimum energy valley between the identified stable states.

### 3.4. Static Calculation

The configurations used in static calculation were taken from the configurations near the free-energy basins obtained from the well-tempered metadynamics. Static calculations employed the same computational level as the AIMD, with the SCF method utilizing diagonalization computations to obtain Molden files for subsequent processing. After the static calculation, Mayer bond orders were calculated using the Multiwfn program [56,57] to quantify the chemical bonding changes. Specifically, Molden files containing the electronic wavefunction were processed to generate a complete inter-atomic bond order matrix. The bond orders corresponding to the target atomic indices (e.g., Fe and O) were then extracted and correlated with their respective bond distances. This integrated workflow ensures that the electronic structure analysis is directly linked to the structural configurations sampled from the AIMD trajectories.” The Interaction Region Indicator (IRI) method [58] was also employed to visualize and quantify weak interactions present within representative configurations. This approach enabled the identification of non-covalent interactions such as hydrogen bonding, van der Waals forces, and steric effects, providing complementary insight into the factors that stabilize intermediate states and govern the energetics of the dissolution process.

## 4. Conclusions

In summary, we employed AIMD and well-tempered metadynamics simulations to construct an atomistic and mechanistic picture of Fe dissolution from the α-Fe_2_O_3_(0001) surface in aqueous environments. The dissolution of Fe is a complex, multi-step, and multi-directional chemical process involving water adsorption, proton exchange, Fe-O bond cleavage, and water molecule diffusion.

Our results demonstrate that the free-energy barrier for complete Fe dissolution is significantly higher than that for surface reconstruction, making defect formation and the appearance of add-atom structures on the surface much more favorable. Hydrogen plays a crucial role throughout the process: it weakens Fe-O bonds and facilitates their rupture while also stabilizing Fe vacancies by occupying them and preventing re-deposition of Fe into its original lattice site.

In contrast, the formation of hydrogen bonds and extended hydrogen-bond networks increases the energetic cost of Fe release. In particular, the formation of a water cage—a structure stabilized by hydrogen bonding and characterized by the exclusion of further proton adsorption—creates a high-energy barrier that makes the final step of dissolution exceedingly difficult. The combination of these effects explains why the α-Fe_2_O_3_(0001) surface is exceptionally stable against complete dissolution, yet prone to surface reconstruction and defect formation, leading to the development of irregular, non-flat surface topographies.

## Data Availability

The data presented in this study are available on request from the corresponding author.

## Supplementary Materials

The following supporting information can be downloaded at: https://www.mdpi.com/article/10.3390/molecules31040748/s1, Figure S1:the overall atomic density of H and O. Figure S2 the the CVs-times plot. Figure S3 The free energy surface as a function of the CN(Fe-Os) or CN(Fe-Ow) every 1 ps (100 Gaussian kernels deposited) along the last 18 ps simulation time before entering the free energy basin of completely dissolution. Figure S4 The IRI analysis of 3 typical transition state configurations with the CVs of (3.0,1.3), (3.0,2.4) and (2.2,2.8) respectly. Figure S5 The stability of the penetrated H atoms in Fe vacancy with Fe migrated or not under a 25ps AIMD. (a) the angle of O-H-O evolution without Fe migrate; (b) the distance of H to opposite O evolution without Fe migrate; (c)the angle of O-H-O evolution with Fe migrate; (d) the distance of H to opposite O evolution with Fe; migrate; (e) the IRI analysis of penetrated H without Fe migrate; (f) the IRI analysis of penetrated H with Fe migrate.
