# Supplementary material for: Atomic-Scale Insights into Surface Reconstruction and Dissolution of Hematite: The Formation of Water Cages and Protonation Effects
Source: Molecules. 2026 Feb 22;31(4):748. doi: 10.3390/molecules31040748 (PMC12942766; doi:10.3390/molecules31040748)
Supplement: Supplementary file 1 [file molecules-31-00748-s001.zip › molecules-4108737-supplementary.pdf]

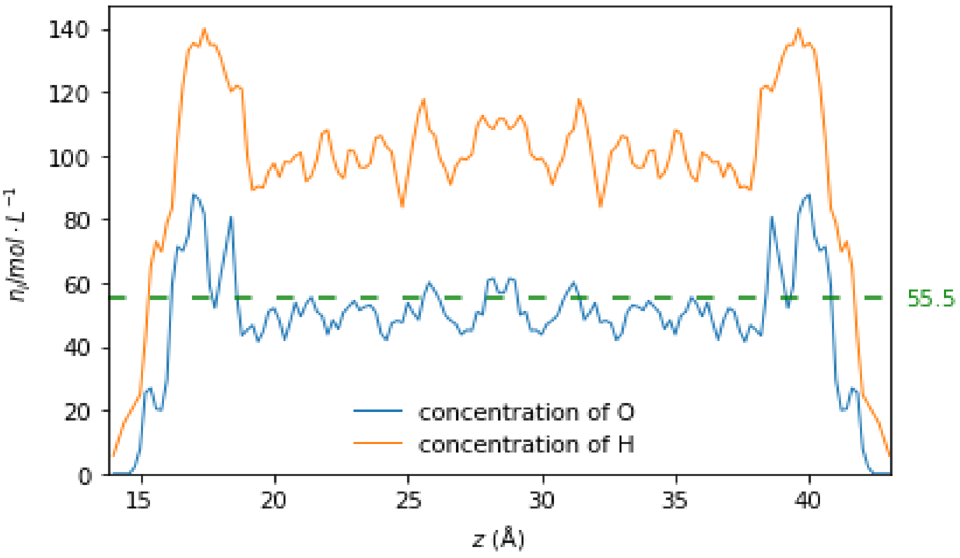

Figure S-1 the overall atomic density of H and O

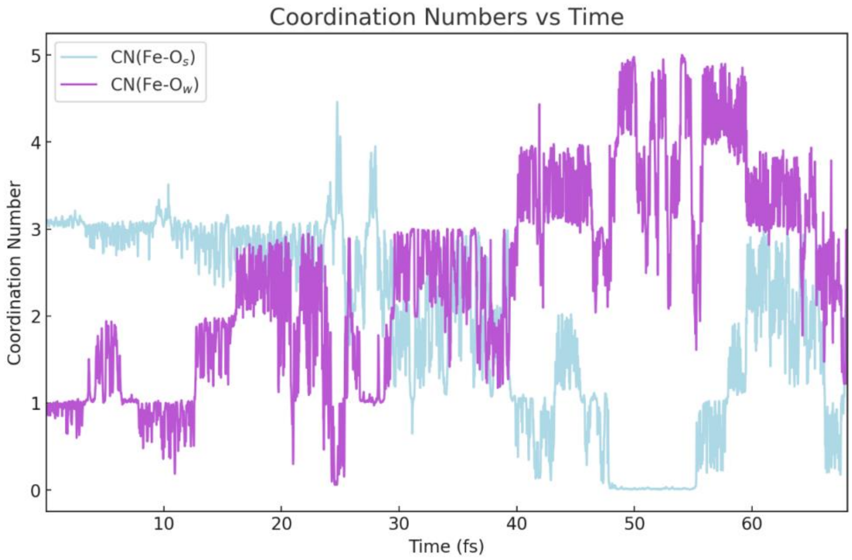

Figure S-2 the the CVs-times plot

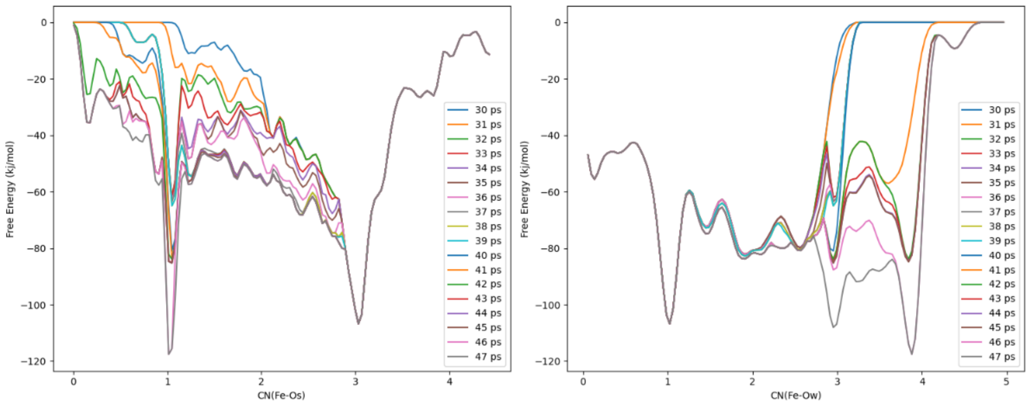

Figure S-3 The free energy surface as a function of the CN(Fe-Os) or CN(Fe-Ow) every 1 ps (100 Gaussian kernels deposited) along the last 18 ps simulation time before entering the free energy basin of completely dissolution.

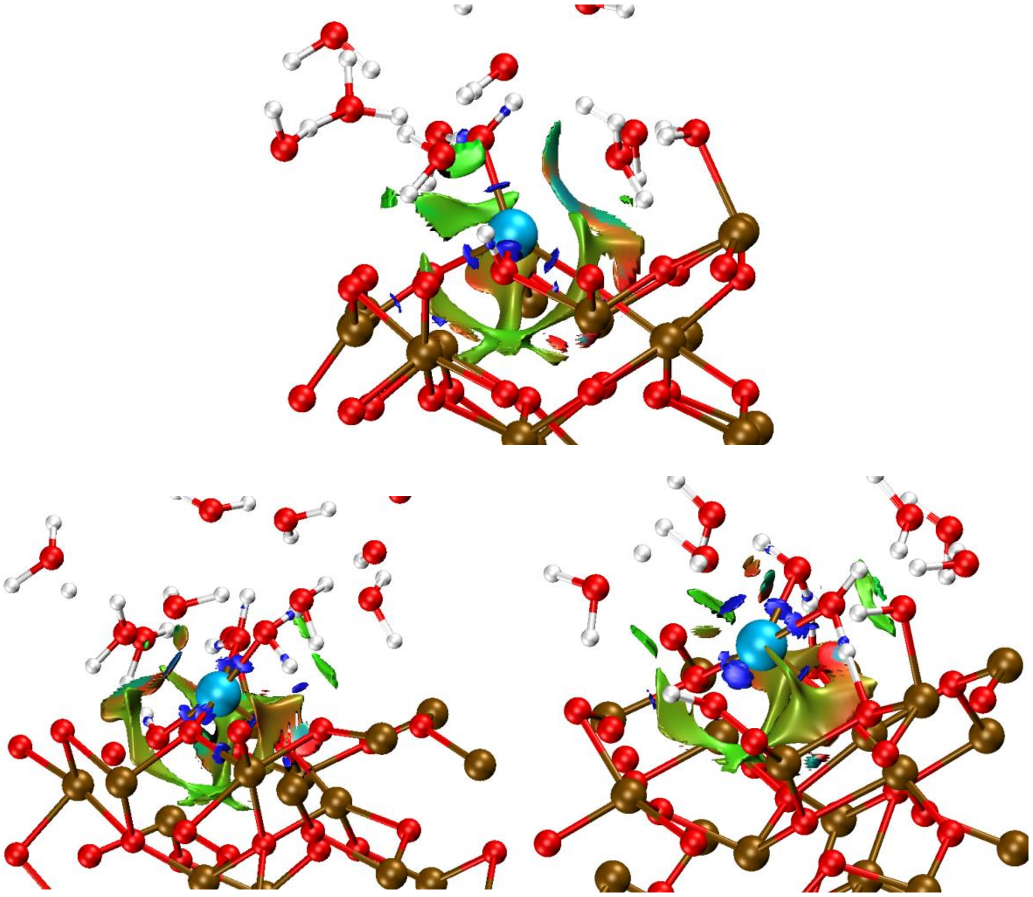

Figure S-4 The IRI analysis of 3 typical transition state configurations with the CVs of (3.0, 1.3), (3.0, 2.4) and (2.2, 2.8) respectly.

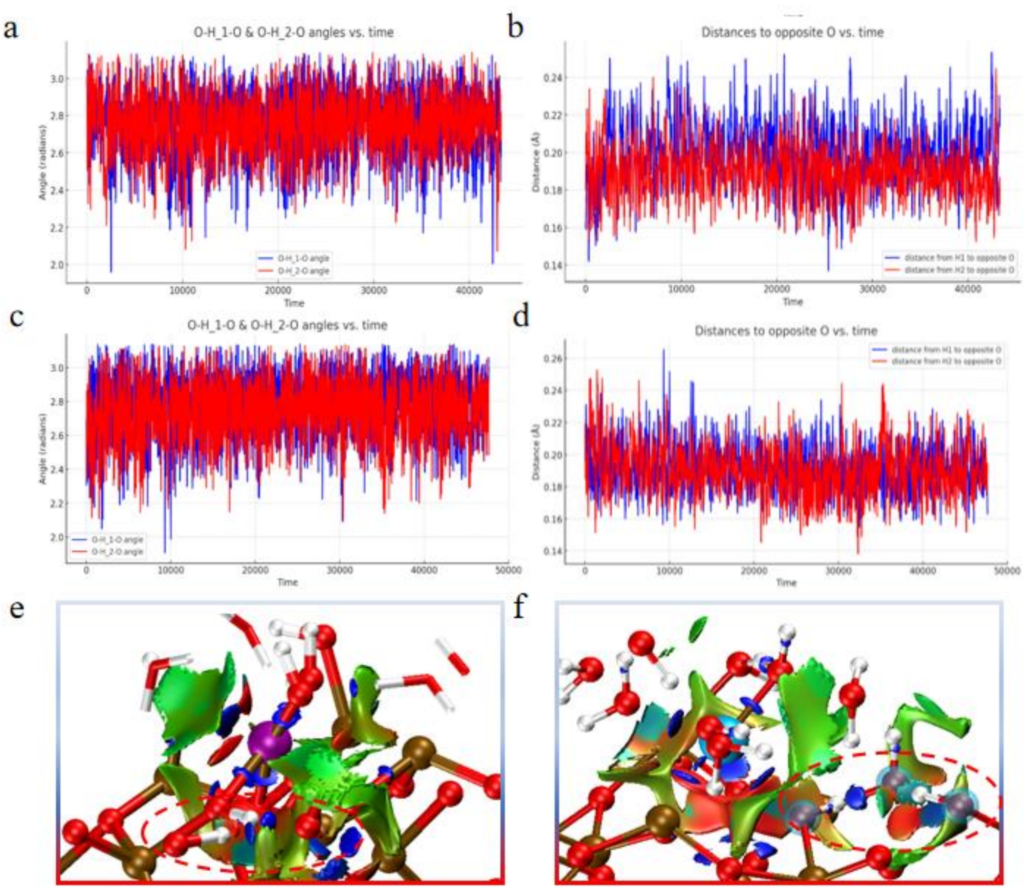

Figure S-5 The stability of the penetrated H atoms in Fe vacancy with Fe migrated or not under a 25ps AIMD. (a) the angle of O-H-O evolution without Fe migrate;(b) the distance of H to opposite O evolution without Fe migrate; (c)the angle of O-H-O evolution with Fe migrate;(d) the distance of H to opposite O evolution with Fe; migrate; (e) the IRI analysis of penetrated H without Fe migrate; (f) the IRI analysis of penetrated H with Fe migrate.
